# Air in the Wrong Place at the Wrong Time: A Case of Pneumomediastinum and Pneumopericardium in the Setting of COVID-19 Disease

**DOI:** 10.7759/cureus.25075

**Published:** 2022-05-17

**Authors:** Priya Patel, Kyle Kelschenbach

**Affiliations:** 1 Internal Medicine, Florida Atlantic University, Boca Raton, USA

**Keywords:** diffuse alveolar damage, intra-alveolar pressure, steroids in covid 19, simple pneumopericardium, spontaneous pneumomediastinum (spm), covid 19

## Abstract

Infection with SARS-CoV-2, commonly referred to as COVID-19 disease, has been noted to involve a systemic inflammatory reaction affecting multiple organ systems. Patients present with a spectrum of symptoms from mild to severe respiratory distress requiring supplemental oxygen and, at times, intubation and mechanical intubation. Pulmonary involvement causes diffuse alveolar wall damage leading to destruction and collapse of the alveolar walls causing air leakage and introduction of the air into the mediastinum, pericardium, and interstitial spaces. We present a case of a 71-year-old patient who presented with respiratory distress requiring supplemental oxygen with subsequent rapid decline and decompensation requiring intubation and mechanical ventilation who was found to have pneumomediastinum and pneumopericardium.

## Introduction

Pneumomediastinum (PM) and pneumopericardium (PP) are conditions where the air has been introduced into the mediastinum and pericardial spaces, respectively; commonly they occur in the setting of chest trauma, cardiothoracic surgery, esophageal rupture, or mechanical ventilation [1].

In individuals with COVID-19 disease, the inflammation and damage to the alveolar walls cause air to dissect into the mediastinum, pericardium, pleural spaces, and subcutaneous tissue, which is also referred to as the alveolar air leak syndrome. Case reports have described these phenomena in patients without prior positive pressure ventilation or mechanical ventilation [2-4].

In most individuals, PM and PP will require close monitoring and will self-resolve, but for the unlucky, the air can expand, leading to hemodynamic instability, causing respiratory compromise requiring surgical decompression [5].

This article was presented as a digital poster at the 2022 Society of Hospital Medicine Florida Chapter Summit Virtual Scientific Abstract Competition.

## Case presentation

The patient is a 71-year-old male with a history of diabetes mellitus and hypertension who presented to the ED via Emergency Medical Services (EMS) from home with complaints of shortness of breath. He had symptoms of an upper respiratory infection 12 days before presentation. Before admission, he endorsed diarrhea and had completed one course of azithromycin. En route to the hospital, EMS noted the oxygen saturation to be 88% on room air, and he was started on 6 liters of a nasal cannula with improvement. He tested positive for SAR-CoV-2, previously unvaccinated. He was started on dexamethasone, remdesivir, and empiric antibiotics. 

**Figure 1:**
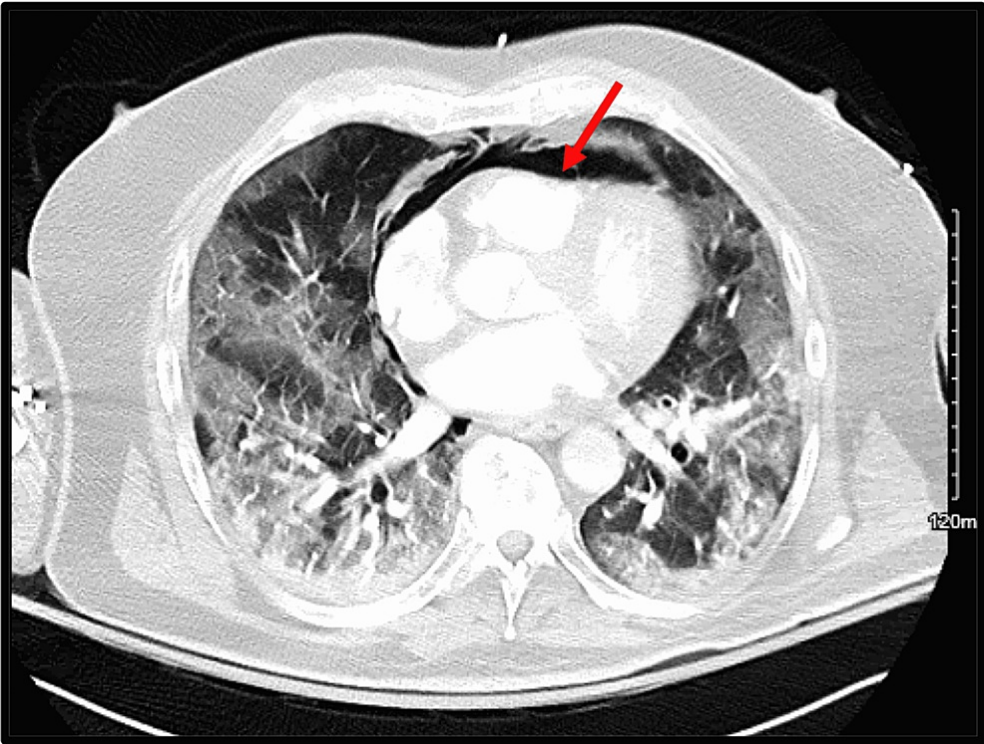
CT chest showing pneumomediastinum (red arrow)

**Figure 2:**
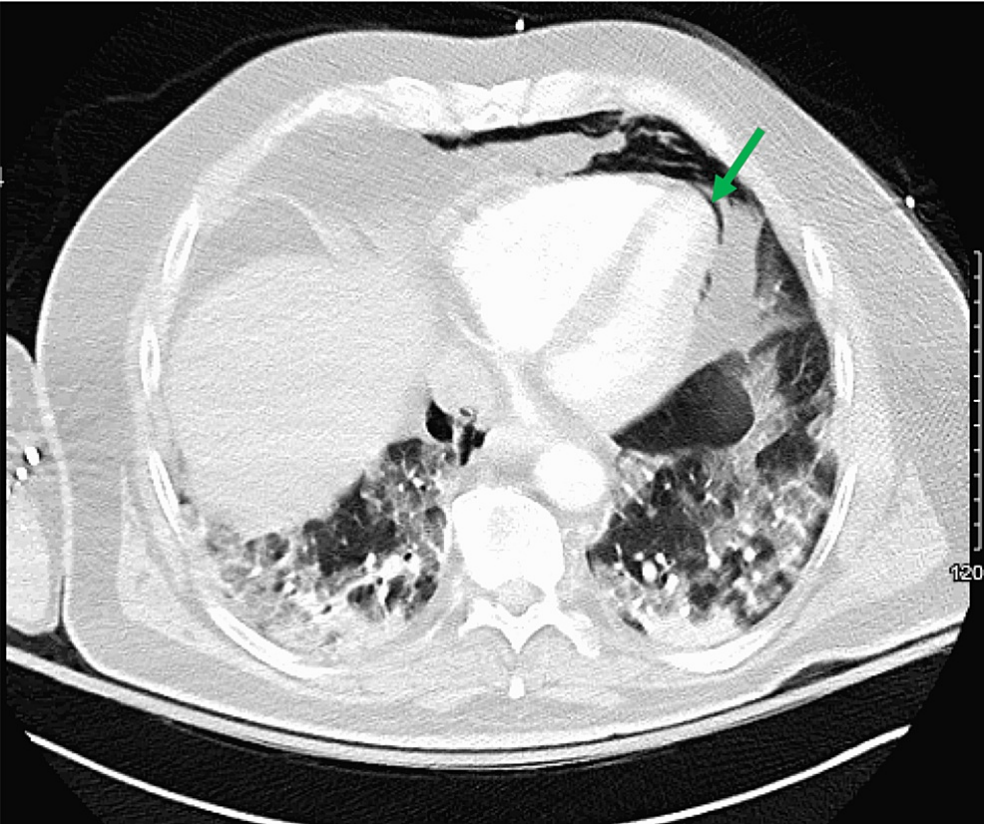
CT chest showing pneumopericardium (green arrow)

Four days into the hospitalization, he had an acute respiratory decompensation with altered mentation, prompting escalation of supplemental oxygen from a 6-liter nasal cannula to a high-flow nasal cannula (HFNC) and a transfer to the intensive care unit (ICU). He was on day 17 of symptoms. His labs were significant for high-sensitivity C-reactive protein 8.6 mg/L (reference range 1-3 mg/L), D-dimer 2.1 mcg/dL (reference <0.5 mcg/dL), and lactic acid 8.9 mg/dL (reference range <2.2 mg/dL). Computed tomography angiography of the chest was negative for pulmonary embolism but did reveal PM and PP (Figure 1 and Figure 2).

His dose of dexamethasone was doubled to 10 mg twice daily, and he was intermittently requiring bilevel positive airway pressure and HFNC. Despite the use of noninvasive therapies, his respiratory status continued to worsen, eventually requiring intubation on day 12 of his hospitalization, day 25 since the onset of symptoms. After intubation, he underwent repeat imaging showing stable PM and PP. He continued to decline, and his ICU stay was complicated by circulatory shock, ischemic hepatopathy, and acute kidney injury. Despite aggressive treatment, he died on hospital day 15.

## Discussion

Spontaneous alveolar air leakage is rare; the incidence was about 12% during the severe acute respiratory syndrome (SARS) outbreak and occurs less than 1% in COVID-19 disease [6]. The pathophysiology is thought to be due to air leakage via diffuse alveolar damage (DAD) leading to an increase in intra-alveolar pressure known as the Macklin effect [7]. This increase in pressure causes air leakage into the interstitial spaces, which can extend into the mediastinum, pleural space, pericardial space, and subcutaneous tissue leading to PM, pneumothorax, PP, and subcutaneous emphysema referred to as alveolar air leak syndrome.

DAD was seen on histopathology in postmortem patients with SARS. The tissue analysis showed exudative features in the first 10-14 days of infection. In a longer disease course, fibrotic features were seen after the 10-14 days period leading to interstitial lung disease and fibrosis [8]. Illness lasting longer would have more features of fibrosis and friable lung tissue predisposing the patient to alveolar leakage.

In addition to DAD, another mechanism thought to increase intra-alveolar pressures includes the presence of cough causing alveolar distension with subsequent rupture. Additionally, steroids that are routinely used in the management of COVID-19 disease and acute respiratory distress syndrome are thought to weaken the pulmonary interstitial tissue, leading to alveolar air leakage [9]. Our patient’s steroid dose was doubled when he acutely decompensated on day 4 of hospitalization and developed a severe, worsening nonproductive cough.

In reviewing the literature, the complications of COVID-19 disease including PM and PP have been reported [2-11]. Additional reports of these complications have been noted to occur in patients before receiving positive pressure ventilation or mechanical ventilation [10,11]. In these case reports, patients developed PM and PP 14-20 days after the onset of symptoms and it was thought to be a feature of severe COVID-19 disease. The patients in the review had elevated lactate dehydrogenase, which is a serum marker that may signify cellular damage [10].

In healthy individuals, an alveolar air leak is self-limited; however, its presence in patients with SARS infection has been demonstrated as a possible predictor of disease severity, higher rates of intubation, and mortality [6]. In the setting of COVID -19, a meta-analysis by Zheng et al. reviewed 13 studies with over 3000 patients. The demographics of those individuals included male gender, age over 65 years, and smoking history, and they found that individuals who met the aforementioned criteria might be at an increased risk of developing a severe illness compounded with chronic comorbidities, which may affect their prognosis. In their meta-analysis, patients who developed a fever along with elevated white blood cell count, creatinine, C-reactive protein, D-dimer, and aspartate aminotransferase could imply progressively worsening COVID-19 disease in conjunction with the presence of alveolar air leak syndrome [12].

## Conclusions

Spontaneous PM and PP are rare complications of COVID-19 disease, which are unique to see outside of the trauma or cardiothoracic surgery setting. This case also encourages early imaging when there is a change in a patient's respiratory status, even in individuals without exposure to positive pressure ventilation. Individuals with alveolar air leak syndrome have a more advanced disease state with higher mortality.
